# Study on resistance mechanisms and molecular epidemiology of carbapenem-resistant *Pseudomonas aeruginosa* to ceftazidime/avibactam in a certain region of China

**DOI:** 10.3389/fcimb.2025.1643755

**Published:** 2025-10-21

**Authors:** Xiaohan Qiao, He Zhang, Yao Xu, Ting Cao, Ruobing Wang, Xinyu Deng, Wei Liang, Lin Zheng

**Affiliations:** ^1^ Department of Clinical Laboratory, The First Affiliated Hospital of Ningbo University, Ningbo, China; ^2^ Department of Medical Laboratory, Bengbu Medical University, Bengbu, China; ^3^ School of Medicine, Ningbo University, Ningbo, China; ^4^ School of Laboratory Medicine and Life Science, Wenzhou Medical University, Wenzhou, China; ^5^ School of Food Science and Engineering, Ningbo University, Ningbo, China; ^6^ Innovative Technologies and Diagnostic and Therapeutic Equipment for Urinary System Diseases, Zhejiang Engineering Research Center, Ningbo, China

**Keywords:** carbapenem-resistant *Pseudomonas aeruginosa*(CRPA), ceftazidime-avibactam (CZA), resistance mechanism, *bla_NDM_* gene, biofilm, efflux pumps, multilocus sequence typing (MLST)

## Abstract

Carbapenem-resistant *Pseudomonas aeruginosa*(CRPA) poses a serious threat in healthcare settings due to its multidrug resistance and high mortality. Although ceftazidime/avibactam (CZA) demonstrates potent activity against CRPA, resistance has emerged.

**Objective:**

This study investigates the epidemiology and molecular mechanisms of CZA resistance in CRPA isolates from Ningbo, China.

**Methods:**

A total of 279 non-duplicate clinical CRPA isolates (2022–2024) were classified as CZA-resistant (CZA-R, n = 68) or CZA-susceptible (CZA-S, n = 211). Carbapenemase genes were detected by PCR, clonality via MLST, biofilm formation by crystal violet assay, and efflux pump expression (*mexA, mexC, mexE, mexY*) via qRT-PCR. WGS was performed on selected isolates.

**Results:**

The CZA resistance rate was 24.37%. Risk factors included recent trauma, prior antibiotic exposure, central venous catheterization, and drainage tube placement (all *p* < 0.05). The CZA-R group showed higher recurrence (13.2% vs. 4.3%, *p* = 0.029) and lower clinical improvement (67.6% vs. 77.3%, *p* = 0.029). *bla_NDM_* prevalence was higher in CZA-R (7.4% vs. 0.5%, *p* = 0.003), and ST1076 was the predominant clone (29.3%), with higher representation in CZA-R (40.0%). Horizontal gene transfer mediated *bla_NDM_* spread. CZA-R isolates exhibited enhanced biofilm formation (*p* < 0.001) and mexA upregulation (2.04-fold, *p* = 0.007).

**Conclusion:**

Our findings indicate a high prevalence of CZA resistance among CRPA isolates in Ningbo, driven by multiple mechanisms including *bla_NDM_* carriage, enhanced biofilm formation, and overexpression of efflux pumps. The dissemination of the high-risk clone ST1076 underscores the need for strengthened infection control measures to curb its spread. These findings provide important insights for optimizing infection control and treatment strategies against CRPA infections in this region.

## 1 Introduction

*Pseudomonas aeruginosa*, a Gram-negative opportunistic pathogen prevalent in healthcare settings, is a leading cause of hospital-acquired infections (Wu et al., 2024) and exhibits broad opportunistic infectivity across human, animal, and plant hosts. Infections caused by *P. aeruginosa* present substantial clinical challenges due to its intrinsic and acquired resistance mechanisms, including efflux pump overexpression (e.g., MexAB–OprM) (Laborda et al., 2021), chromosomal mutations (Leilei et al., 2024), and robust biofilm formation (Sharma et al., 2023). Furthermore, *P. aeruginosa* thrives under hospital-relevant stress conditions such as nutrient limitation and oxidative stress (Sharma et al., 2019), complicating infection control efforts. Its capacity to form persistent biofilms on medical devices (Muhammad et al., 2020) further elevates the risk of device-related infections and treatment failures.

Over the past decade, the global spread of carbapenem resistance has contributed significantly to the increasing prevalence of *P. aeruginosa*. In response, the World Health Organization (WHO) has classified carbapenem-resistant *P. aeruginosa*(CRPA) as a critical-priority pathogen (Sati et al., 2025). Key CRPA resistance mechanisms encompass carbapenemase acquisition, loss of the outer membrane porin OprD, efflux pump overexpression, and hyperproduction of chromosomal AmpC β-lactamases (Yuexing et al., 2022).

Ceftazidime/avibactam (CZA) has emerged as a valuable therapeutic option for CRPA infections, demonstrating potent activity against strains producing extended-spectrum β-lactamases (ESBLs), AmpC enzymes, and class A carbapenemases such as KPC (Matesanz and Mensa, 2021; Chuanfu et al., 2024). Approved internationally since 2016 for complicated intra-abdominal infections, urinary tract infections, hospital-acquired pneumonia, and ventilator-associated pneumonia—particularly when treatment options are limited—CZA was specifically endorsed in China in 2019 for the treatment of adult patients with *P. aeruginosa* infections lacking alternatives (Hu et al., 2023). Accumulating clinical evidence supports the efficacy of CZA against multidrug-resistant *P. aeruginosa* (Laura et al., 2022).

However, emerging studies indicate that rising clinical use of CZA has been accompanied by increasing resistance rates among *P. aeruginosa* strains (A et al., 2023). The emergence of CZA-resistant CRPA constitutes a serious threat to effective patient management (Dilip et al., 2023), yet the molecular mechanisms and regional epidemiological characteristics of such resistance remain inadequately elucidated.

Therefore, this study was conducted to characterize the epidemiology and resistance mechanisms of CZA-resistant CRPA isolates collected from the First Affiliated Hospital of Ningbo University between January 2022 and October 2024. Our findings aim to support the development of strategies to counter the emergence of CZA resistance and provide an evidence base for targeted antimicrobial therapy.

## 2 Materials and methods

### 2.1 Bacterial isolate collection

A total of 279 clinical isolates of CRPA were selected from the –80 °C strain repository of The First Affiliated Hospital of Ningbo University between January 2022 and October 2024. The inclusion criteria were as follows: (1) patients aged ≥ 18 years; (2) infection confirmed to be caused by CRPA; (3) isolates obtained from normally sterile sites, urine, lower respiratory tract, or wounds. Exclusion criteria included: (1) hospital stay less than 3 days; (2) incomplete CRPA-related clinical or microbiological data; (3) repeated isolates from the same patient within 30 days (Chen et al., 2024). This study was approved by the Ethics Committee of The First Affiliated Hospital of Ningbo University.

### 2.2 Experimental methods

#### 2.2.1 Strain identification

All selected strains were inoculated onto Columbia blood agar and incubated at 37°C for 24 hours according to the guidelines of the Clinical and Laboratory Standards Institute (CLSI) M100, 35th edition (CLSI, 2025). Species identification of *P. aeruginosa*was confirmed using the VITEK 2 Compact automated system (bioMérieux, France).

#### 2.2.2 Antimicrobial susceptibility testing

Antimicrobial susceptibility testing was performed using both the disk diffusion and broth microdilution methods in strict accordance with CLSI guidelines (CLSI, 2024a; CLSI, 2024b). Interpretation of results was based on CLSI M100, 35th edition (CLSI, 2025), with the exception of colistin, for which results were interpreted following international consensus guidelines (Tsuji et al., 2019).

CRPA Screening: Isolates were defined as CRPA based on resistance to at least one of the following carbapenems: imipenem, meropenem, doripenem, or ertapenem (Wu et al., 2024). Screening was performed using imipenem (IPM) disk diffusion, with zone diameters of ≤15 mm indicating resistance (Muhammad et al., 2020).

CZA Susceptibility Testing: Susceptibility to CZA was determined via disk diffusion. Zone diameters ≥21 mm were classified as susceptible, and ≤20 mm as resistant (Muhammad et al., 2020). Based on these results, isolates were categorized into CZA-susceptible (CZA-S) and CZA-resistant (CZA-R) groups.

Comprehensive AST: Additional susceptibility profiling was conducted using the VITEK 2 Compact system (bioMérieux, France) with the appropriate AST cards. *P. aeruginosa* ATCC^®^ 27853 was used as the quality control strain for all susceptibility testing procedures. The reference ranges for antimicrobial susceptibility testing results by VITEK 2 are provided in **Supplementary Table S1**.

#### 2.2.3 Clinical data collection

Relevant clinical data were extracted from the hospital’s Electronic Medical Record (EMR) system for all included isolates.

#### 2.2.4 Carbapenemase gene detection

Genomic DNA was extracted from isolates using a rapid boiling method (Dallenne et al., 2010). The presence of eight major carbapenemase genes (*bla_KPC_、bla_GES_、bla_NDM_、bla_VIM_、bla_IMP_、bla_SPM_、bla_PDC_、bla_OXA-50_*) was assessed by PCR with specific primers (**Table 1**). Amplified products were visualized via gel electrophoresis.

**Table 1:** Primer sequences of genotyping of the carbapenemase.

Gene name	Direction	Primers (5’-3’)	Product size
*bla_PDC_*	Forward Reverse	CTGCCTGTGCGGCATCGCCG TCCTGGGCCAGGGCATAG	300 bp
*bla_GES_*	Forward Reverse	ATGCGCTTCATTCACGCAC CTATTTGTCCGTGCTCAGG	846 bp
*bla_OXA-50_*	Forward Reverse	ATGCGCCCTCTCCTCTTCAG GTAACCCAGGCGCGAGACAT	420 bp
*bla_KPC_*	Forward Reverse	TAGTTCTGCTGTCTTGTCTC GTGCTTGTCATCCTTGTTAG	920 bp
*bla_IMP_*	Forward Reverse	GGCTTATCTAATTGACACTCC TAACCGCCTGCTCTAATG	277 bp
*bla_NDM_*	Forward Reverse	CGCTTCCAACGGTTTGAT GCTCATCACGATCATGCT	984 bp
*bla_VIM_*	Forward Reverse	GCGGAGATTGAGAAGCAA GCAGCACCAGGATAGAAG	380 bp
*bla_SPM_*	Forward Reverse	TGTTTGTTGCTCGTTGCG CATGCCTTCACATTGGCATCTC	786 bp

#### 2.2.5 Multilocus sequence typing

Multilocus sequence typing was performed according to the established *P. aeruginosa* MLST scheme (Curran et al., 2004). Seven housekeeping genes (*acsA, aroE, guaA, mutL, nuoD, ppsA, and trpE*; primer sequences listed in **Table 2**) were amplified by PCR. The amplified fragments were sequenced, and the resulting sequences were compared against the PubMLST database (https://pubmlst.org/) to determine the corresponding allele numbers. The unique Sequence Type (ST) for each isolate was assigned by submitting the sequence of the seven allele numbers to the PubMLST database.

**Table 2:** MLST housekeeping genes amplification and sequencing primers.

Gene name	Direction	Primers (5’-3’)	Product size
*acsA*	Forward Reverse	ACCTGGTGTACGCCTCGCTGAC GACATAGATGCCCTGCCCCTTGAT	779 bp
*aroE*	Forward Reverse	TGGGGCTATGACTGGAAACC TAACCCGGTTTTGTGATTCCTACA	755 bp
*guaA*	Forward Reverse	CGGCCTCGACGTGTGGATGA GAACGCCTGGCTGGTCTTGTGGTA	844 bp
*mutL*	Forward Reverse	CCAGATCGCCGCCCGGTGAGGGTG CAGGGTGCCATAGAGGAAGTC	822 bp
*nuoD*	Forward Reverse	ACCGCCACCCCGTACTG TCTCGCCCATCTTGACCA	839 bp
*ppsA*	Forward Reverse	GGTCGCTCGGTCAAGGTAGTGG GGGTTCTCTTCTTCCGGGCTCGTAG	901 bp
*trpE*	Forward Reverse	GCGGCCCAGGGT CGTGAG CCCGGCGCTTGTTGATGGTT	760 bp

#### 2.2.6 Biofilm formation assay

Biofilm formation was evaluated using the crystal violet (CV) staining method as previously described (Zhang et al., 2024). Sixty-eight CZA-R and 68 CZA-S isolates were randomly selected. Overnight cultures grown in Lysogeny Broth (LB) at 37 °C with shaking at 200 rpm were diluted 1:100 in fresh LB to an optical density at 570 nm (OD_570_) of 1.0–1.5. Then, 200 µL of each diluted culture was transferred into a 96-well polystyrene plate and incubated statically at 37°C for 24–48 hours. After incubation, the medium was discarded, and the wells were gently washed twice with phosphate-buffered saline (PBS), fixed with 9% methanol for 15 minutes, air-dried, stained with 1% crystal violet for 5 minutes, and thoroughly rinsed. The bound dye was dissolved in 33% glacial acetic acid and incubated at 37°C for 30 minutes. The optical density at 570 nm (OD_570_) was measured using a SpectraMax ID3 microplate reader (Molecular Devices, USA), with three replicates per isolate.

The cutoff value (ODc) was defined as the mean optical density of the negative controls plus three standard deviations. Biofilm formation was categorized as follows: OD ≤ ODc = non-biofilm former (–); ODc < OD ≤ 2×ODc = weak biofilm former (+); 2×ODc < OD ≤ 4×ODc = moderate biofilm former (++); OD > 4×ODc = strong biofilm former (+++) (T et al., 2025).

#### 2.2.7 Efflux pump gene expression analysis

Twenty-four CZA-R and 24 CZA-S isolates were randomly selected for quantification of efflux pump gene expression (*mexA, mexC, mexE, mexY*) as previously described (Li et al., 2023). Total RNA was extracted using the Magen Bacterial RNA Extraction Kit (Magen, China). Complementary DNA (cDNA) was synthesized using the ABMGood One-Step RT MasterMix (Applied Biological Materials Inc., Canada). Quantitative reverse transcription PCR (qRT-PCR) was performed using SYBR Green Master Mix (CWBIO, China) with *rpoD* as the reference gene. Primer sequences were derived from previously published studies (Horna et al., 2018; Li et al., 2024), with detailed information provided in **Supplementary Table S2**. Each 20 µL reaction mixture contained 2 µL cDNA, 0.6 µL each of forward and reverse primers, 10 µL Master Mix, and 6.8 µL ddH_2_O. The thermal cycling conditions were as follows: 95°C for 2 minutes; 40 cycles of 95°C for 10 seconds and 60°C for 30 seconds; followed by a melt curve analysis. All reactions were performed in triplicate, and relative gene expression was calculated using the 2^–ΔΔCt^ method.

#### 2.2.8 Whole-genome sequencing

Genomic DNA was extracted from eight representative ST1076 isolates (four CZA-R and four CZA-S) using the TIANGEN Bacterial Genomic DNA Kit (TIANGEN, China). Sequencing was performed on an Illumina NovaSeq 6000 platform (2×150 bp) by Weishu Biotechnology Co., Ltd. (Hangzhou, China). *De novo* assembly was conducted using SPAdes, and genome annotation was performed with Prokka.

### 2.3 Statistical analysis

All statistical analyses in this study were performed using SPSS software (version 27.0; IBM, Chicago, IL, USA). A significance threshold of α = 0.05 was applied for all tests. Categorical variables—including clinical parameters (risk factors, disease associations, patient outcomes), antimicrobial resistance profiles, and carbapenemase gene distributions—were compared between groups using the chi-square test (when expected frequencies were ≥5) or Fisher’s exact test (when expected frequencies were <5). Continuous variables were analyzed using the Mann–Whitney U test for non-normally distributed data (e.g., biomarker levels, biofilm formation quantified by OD values) and the independent samples t-test for normally distributed data (e.g., expression of efflux pump genes such as mexE). GraphPad Prism (version 8; La Jolla, CA, USA) was used to generate figures, while statistical results were analyzed using SPSS 27. All p-values were two-tailed, and results were considered statistically significant at *p* < 0.05.

## 3 Results

### 3.1 Sample collection of CRPA strains

**Figure 1:**
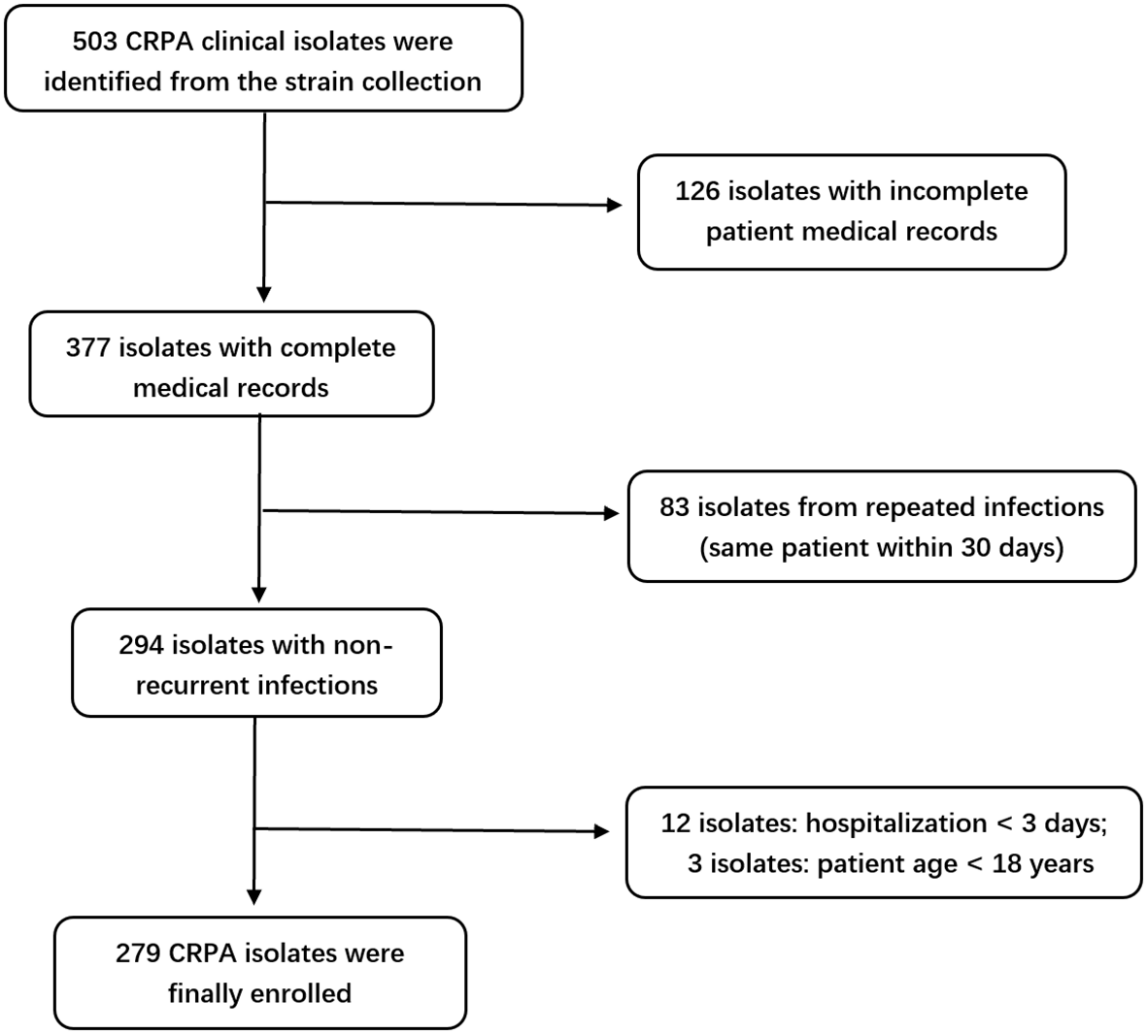
Flowchart of the screening process for 279 CRPA isolates.

**Figure 2:**
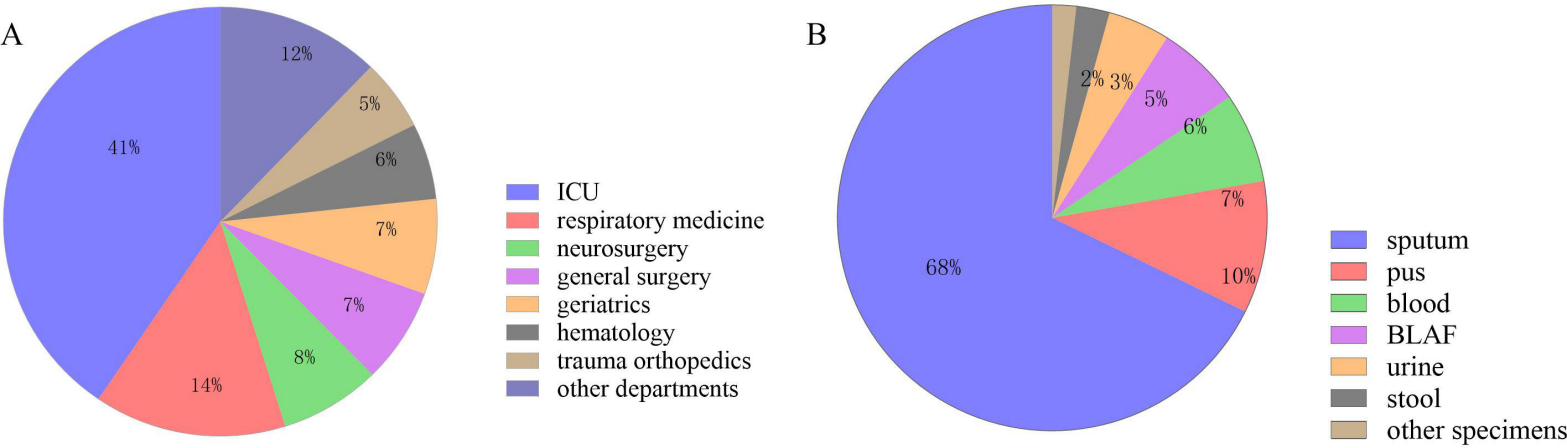
Sample collection of 279 CRPA strains. **(A)** Departmental Distribution of 279 CRPA Isolates; **(B)** Specimen Type Distribution of 279 CRPA Isolates.

A total of 279 CRPA clinical isolates meeting the inclusion criteria were ultimately included in this study (**Figure 1**). The isolates were predominantly obtained from the intensive care unit (ICU) (113 isolates, 40.50%), followed by the Department of Respiratory Medicine (40 isolates, 14.34%), and the Department of Neurosurgery (21 isolates, 7.53%), among other clinical units (**Figure 2**). Respiratory specimens constituted the majority of sample types (207 isolates, 74.19%), including sputum (189 samples) and bronchoalveolar lavage fluid (BALF, 18 samples). The remaining isolates were derived from pus (28 isolates, 10.04%), blood (19 isolates, 6.81%), and other sources (**Figure 2**).

### 3.2 Clinical parameter analysis

#### 3.2.1 Cohort selection and grouping

Among the 279 CRPA isolates subjected to ceftazidime-avibactam (CZA) susceptibility testing, 24.37% (68/279) were identified as resistant (CZA-R group), while 75.63% (211/279) were susceptible (CZA-S group).

#### 3.2.2 Risk factors for infection

Multivariate analysis identified the following independent risk factors significantly associated with CZA resistance (*p* < 0.01): recent trauma history (within ≤3 months; 23.5% in CZA-R vs. 10.0% in CZA-S), prior antibiotic exposure (88.2% vs. 37.9%), presence of a central venous catheter (80.9% vs. 36.5%), and indwelling drainage tubes (66.2% vs. 37.4%) (**Table 3**). In contrast, factors such as diabetes, malignancy, and mechanical ventilation showed no significant association with CZA resistance (*p* > 0.05) (**Table 3**).

**Table 3:** Comparison of risk factors for infection between CZA-resistant and CZA-susceptible CRPA isolates [n(%)].

Risk factor	CZA-R group (n=68)	CZA-S group (n=211)	χ²	*P* -value
History of trauma (≤3 months)	16 (23.5)	21 (10.0)	8.241	0.004
Prior antibiotic use	60 (88.2)	80 (37.9)	52.089	<0.001
Central venous catheterization	55 (80.9)	77 (36.5)	40.650	<0.001
Indwelling drainage tube†	45 (66.2)	79 (37.4)	17.198	<0.001
Diabetes	15 (22.1)	62 (29.4)	1.381	0.240
Long-term glucocorticoid use	1 (1.5)	6 (2.8)	0.034	0.854
Granulocytopenia	3 (4.4)	6 (2.8)	0.058	0.809
Chronic liver disease	4 (5.9)	9 (4.3)	0.048	0.826
Chronic kidney disease	1 (1.5)	12 (5.7)	1.219	0.270
Hemodialysis	3 (4.4)	14 (6.6)	0.141	0.708
Malignancy	16 (23.5)	53 (25.1)	0.070	0.792
Invasive procedure (≤3 months)	22 (32.4)	78 (37.0)	0.476	0.490
History of surgery (≤3 months)	22 (32.4)	87 (41.2)	1.703	0.192
History of hospitalization (≤3 months)	16 (23.5)	55 (26.1)	0.174	0.676
ICU admission (≤3 months)	6 (8.8)	12 (5.7)	0.838	0.360
Smoking	5 (7.4)	14 (6.6)	0.042	0.838
Immunocompromised status	60 (88.2)	179 (84.8)	0.484	0.486
Mechanical ventilation	35 (51.5)	105 (49.8)	0.060	0.807
Previous CRPA infection history	22 (32.4)	57 (27.0)	0.722	0.395
Cytochrome-producing phenotype	58 (85.3)	181 (85.8)	0.010	0.920

†Including urinary catheter, nasogastric tube, and abdominal drainage tube; We employed the Chi-square test (χ² test) to perform a statistical analysis comparing the infection risk factors between patients infected 68 CZA-R and 211 CZA-S CRPA isolates.

#### 3.2.3 Disease association analysis

The incidence of respiratory failure (60.3% vs. 39.8%, *p* = 0.003), skin and soft tissue infections (7.4% vs. 1.4%, *p* = 0.033), and fractures (17.6% vs. 5.7%, *p* = 0.002) was significantly higher in the CZA-R group than in the CZA-S group. No significant differences were observed in the incidence of pneumonia, sepsis, urinary tract infections, or other diseases between the two groups (*p* > 0.05) (**Table 4**).

**Table 4:** Comparison of disease characteristics between CZA-resistant and CZA-susceptible CRPA groups [n(%)].

Disease	CZA-R group (n=68)	CZA-S group (n=211)	χ²	*P* -value
Respiratory failure	41 (60.3)	84 (39.8)	8.725	0.003
Skin/soft tissue infection‡	5 (7.4)	3 (1.4)	4.540	0.033
Fracture	12 (17.6)	12 (5.7)	9.356	0.002
Pneumonia	32 (47.1)	72 (34.1)	3.680	0.055
Severe pneumonia	17 (25.0)	43 (20.4)	0.650	0.420
Sepsis	10 (14.7)	30 (14.2)	0.010	0.920
Urinary tract infection	4 (5.9)	13 (6.2)	<0.001	>0.999

‡Includes abscess and wound infection; We employed the Chi-square test to perform a statistical analysis comparing the infection disease characteristics between patients infected with 68 CZA-R and 211 CZA-S CRPA isolates.

#### 3.2.4 Biomarker analysis

No significant differences were observed between the two groups regarding age, levels of inflammatory biomarkers (white blood cell count, procalcitonin, C-reactive protein, serum amyloid A, interleukin-6), or length of hospital stay (p> 0.05) (**Table 5**).

**Table 5:** Comparison of clinical parameters between patients with CZA-resistant and CZA-susceptible CRPA infections [Median (IQR)].

Clinical parameter	CZA-R group (n=68)	CZA-S group (n=211)	Z statistic	*P* -value
Age (years)	73.0 (65.25–81.75)	72.0 (58.00–81.00)	-0.985	0.324
White blood cell count (×10^9^/L)	12.0 (6.93–17.82)	9.9 (6.50–15.70)	-1.071	0.284
Neutrophil percentage (%)	80.55 (74.15–87.53)	80.00 (71.40–87.30)	-0.688	0.492
Procalcitonin (ng/mL)	0.46 (0.17–2.62)	0.82 (0.16–3.63)	-0.470	0.638
Serum amyloid A (IU/L)	235.90 (60.80–356.98)	203.70 (36.90–562.10)	-0.076	0.940
C-reactive protein (mg/L)	34.13 (13.35–108.28)	28.56 (11.37–90.40)	-1.176	0.240
Interleukin-6 (pg/mL)	65.61 (20.25–231.39)	67.87 (20.99–259.17)	-0.242	0.809
Length of hospital stay (days)	20.5 (12–43.5)	27 (15–55)	-1.504	0.133

We employed the Mann-Whitney U test to compare clinical parameters between patients infected with 68 CZA-R and 211 CZA-S CRPA isolates.

#### 3.2.5 Patient outcome analysis

A. Infection-related outcomes: Significant differences were observed in the distribution of clinical outcomes (clinical improvement, recurrence, death) between the two groups (χ² = 7.058, *p* = 0.029). The clinical improvement rate was significantly lower in the CZA-R group (67.6%) compared to the CZA-S group (77.3%), while the recurrence rate was significantly higher in the CZA-R group (13.2% vs. 4.3%). No significant difference was found in mortality rates between the two groups (19.1% vs. 18.5%, *p* > 0.05) (**Table 6**).

**Table 6:** Comparison of clinical and hospitalization outcomes between patients with CZA-resistant and CZA-susceptible CRPA infections [n(%)].

Outcome category	Outcome	CZA-R group (n=68)	CZA-S group (n=211)	χ²	*P* -value
Infection-related outcomes	Clinical improvement	46 (67.6)	163 (77.3)	7.058	0.029
	Recurrence	9 (13.2)	9 (4.3)		
	Death	13 (19.1)	39 (18.5)		
Hospitalization outcomes	Discharge	38 (55.9)	119 (56.4)	0.014	0.993
	Death	13 (19.1)	39 (18.5)		
	Continued treatment	17 (25.0)	53 (25.1)		

We employed the Chi-square test to compare clinical outcomes and hospitalization outcomes between patients infected with 68 CZA-R and 211 CZA-S CRPA isolates.

B. Hospitalization outcomes: No significant differences were observed in hospitalization outcomes (discharge, death, continued treatment) between the groups (χ² = 0.014, *p* = 0.993) (**Table 6**).

### 3.3 Antimicrobial susceptibility testing

#### 3.3.1 Overall resistance profile

**Figure 3:**
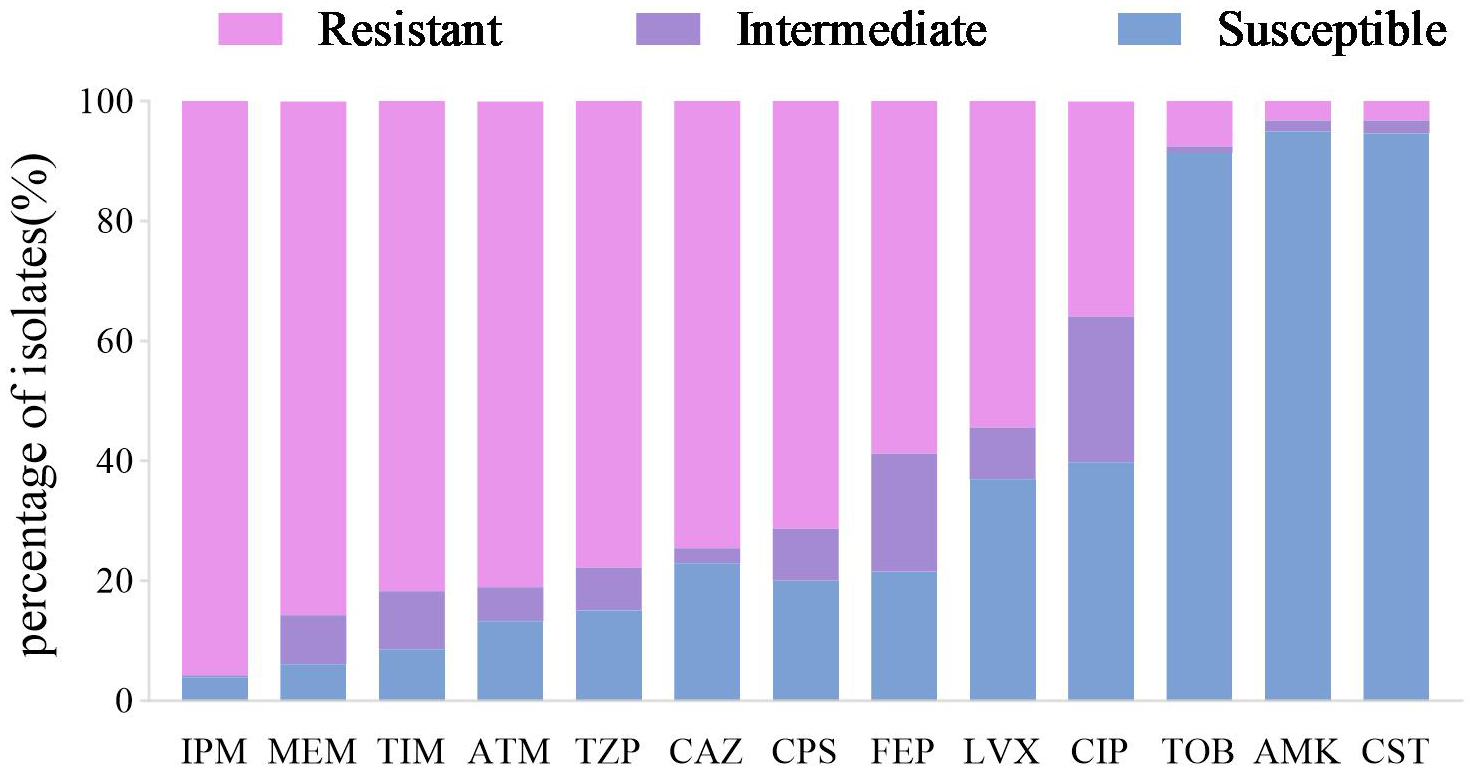
Antimicrobial susceptibility profile of 279 CRPA clinical isolates. Abbreviations: IPM, imipenem; MEM, meropenem; TIM, ticarcillin-clavulanate; ATM, aztreonam; TZP, piperacillin-tazobactam; CAZ, ceftazidime; CPS, cefoperazone-sulbactam; FEP, cefepime; LVX, levofloxacin; CIP, ciprofloxacin; TOB, tobramycin; AMK, Amikacin;CST, Colistin.

Antimicrobial susceptibility testing against 13 antimicrobial agents from 7 classes was performed on all 279 CRPA clinical isolates. The highest resistance rate was observed against imipenem (95.70%), followed by meropenem (85.66%) and ticarcillin/clavulanic acid (81.72%). The lowest resistance rates were identified for amikacin (3.23%, 9/279) and colistin (3.23%, 9/279) (details shown in **Figure 3**). The overall prevalence of multidrug resistance (MDR) was 86.02%, with a significantly higher MDR rate in the CZA-R group compared to the CZA-S group (94.12% vs. 83.41%; χ² = 4.901, *p* = 0.027). For detailed antimicrobial resistance data, see **Supplementary Table S3**; for multidrug resistance (MDR) results, refer to **Supplementary Table S4**.

#### 3.3.2 Intergroup resistance differences

**Figure 4:**
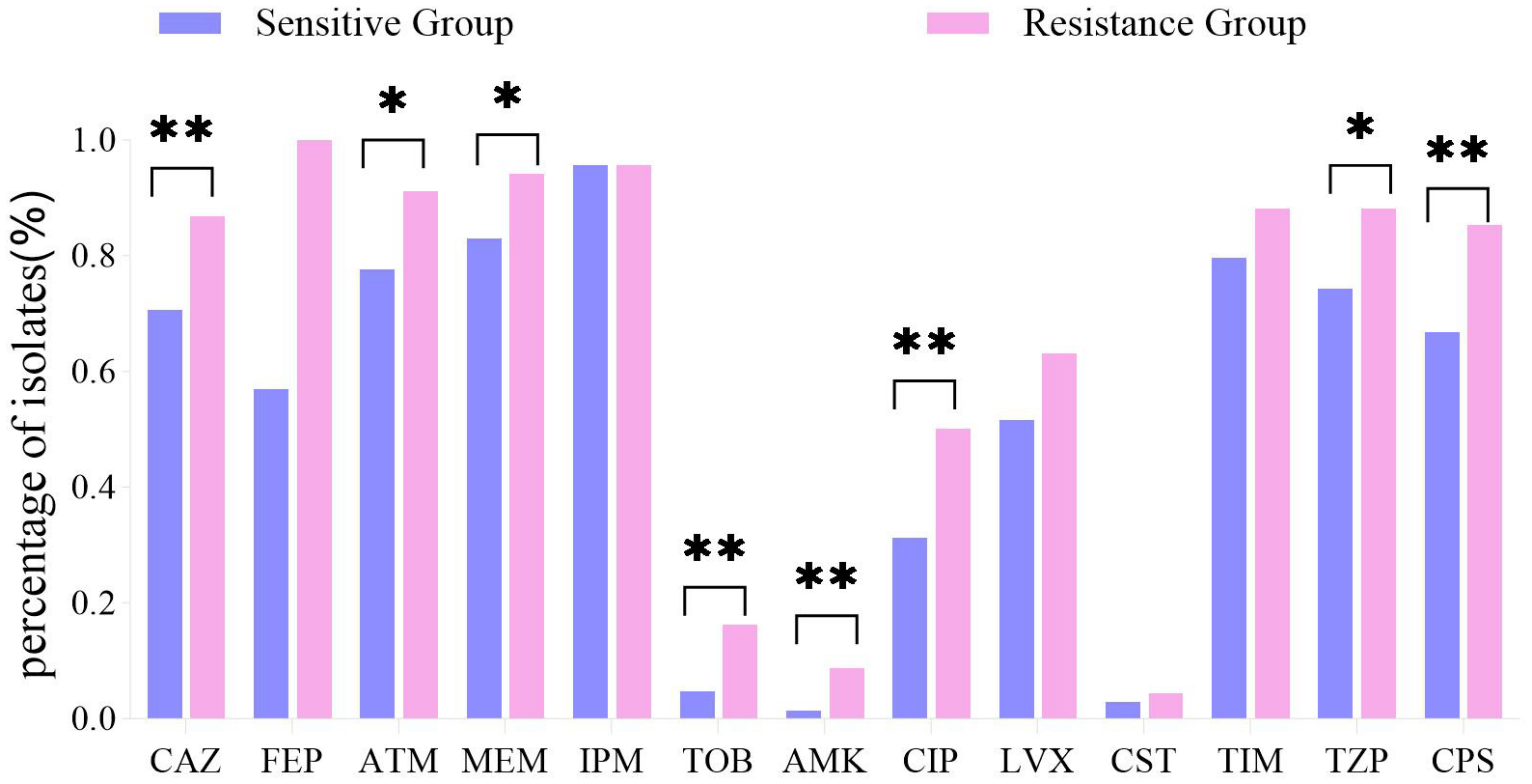
Antibiotic resistance profiles: CZA-R vs. CZA-S CRPA isolates. Comparison of antibiotic resistance profiles between 68 CZA-R and 211 CZA-S CRPA.Statistical comparison was performed using the Chi-square test. SPSS 27.0. p  < <.0. (marked *) was defined as a significant difference, and p < 0.001 (marked as **) was deemed highly significan.

CZA-R isolates exhibited significantly higher resistance rates to most tested antimicrobial agents (*p* < 0.05 for all comparisons), including ceftazidime, aztreonam, meropenem, tobramycin, amikacin, ciprofloxacin, piperacillin/tazobactam, and cefoperazone/sulbactam (**Figure 4**).

### 3.4 Molecular characteristics

#### 3.4.1 Carbapenemase genes

**Figure 5:**
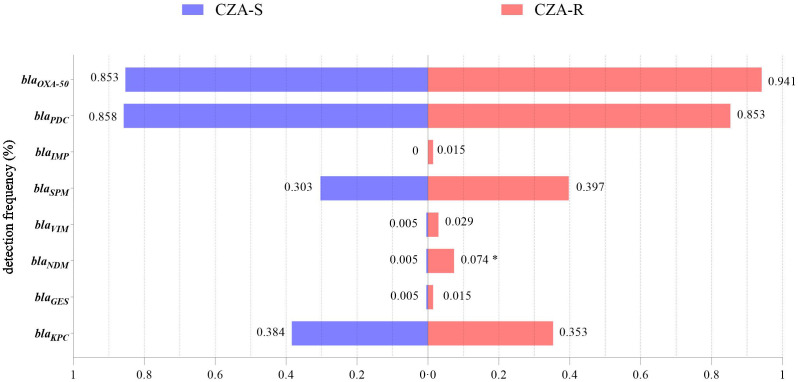
Detection of carbapenemase genes in 68 CZA-R and 211 CZA-S CRPA isolates.Statistical comparison was performed using the Chi-square test (for expected frequencies ≥5) or Fisher’s exact test (for expected frequencies <5). SPSS 27.0. p  < <.0. (marked *) was defined as a significant difference.

The detection rate of *bla_NDM_* was significantly higher in the CZA-R group than in the CZA-S group (7.35% vs. 0.47%; χ² = 8.527, *p* = 0.003), indicating its role as a key molecular marker of CZA resistance (**Figure 5**). No significant differences were observed in the distribution of other carbapenemase genes between the two groups (*p* > 0.05).

#### 3.4.2 Multilocus sequence typing

**Figure 6:**
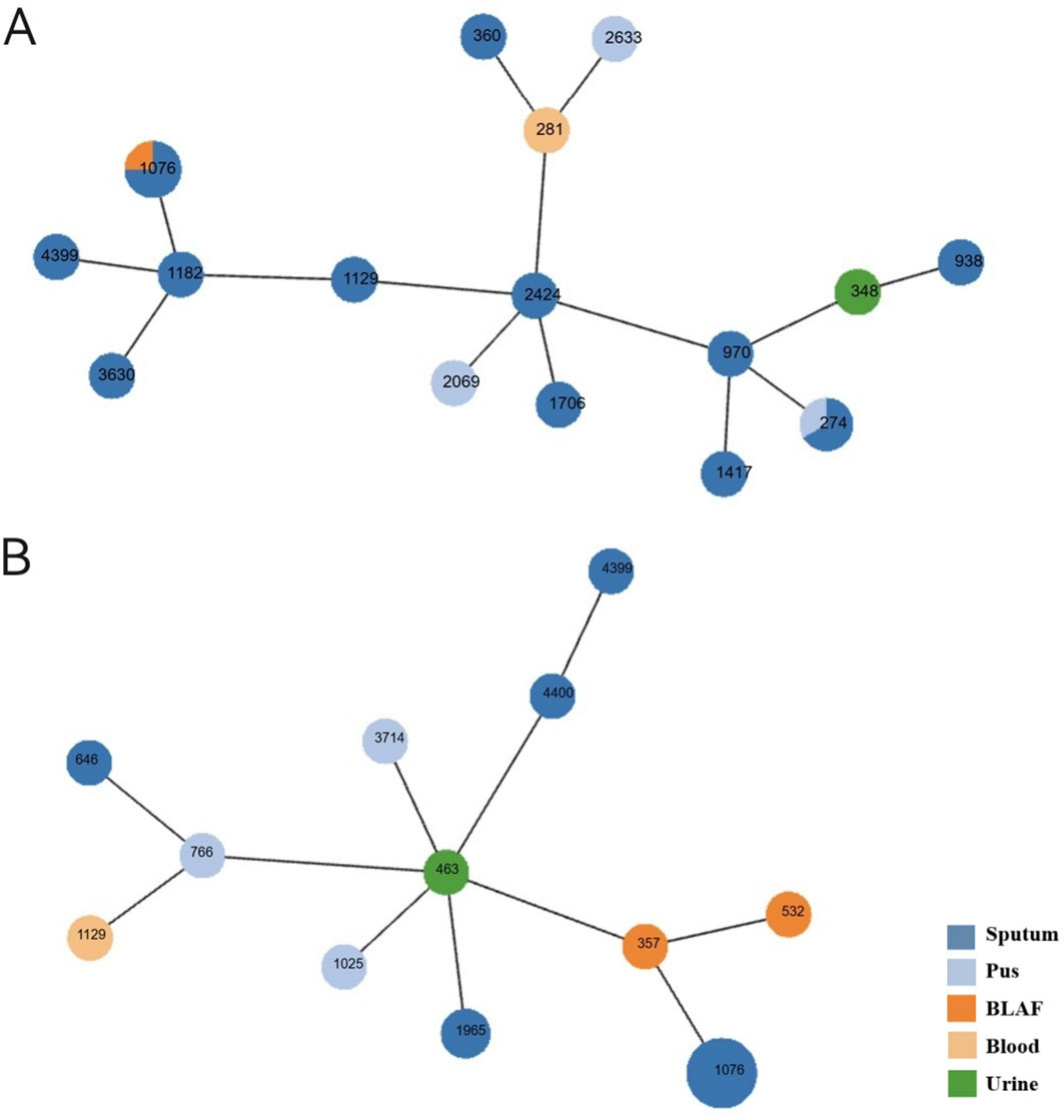
Minimum spanning tree of CRPA MLST profiles. **(A)** CZA-sensitive isolates **(B)** CZA-resistant isolates Each node represents a sequence type (ST). Node size is proportional to the number of isolates within that ST. Node colors indicate isolation sources. Connecting lines reflect genetic distance between STs.

Multilocus sequence typing was performed on 41 CRPA isolates, revealing high genetic diversity with a total of 25 distinct sequence types (STs) identified. ST1076 was the predominant epidemic lineage, accounting for 29.3% (12/41) of the isolates. It was also the dominant ST in both the CZA-R (8/20, 40.0%) and CZA-S (4/21, 19.0%) groups. Other STs (including ST274, ST1129, ST1965, ST4399, etc.) were detected at lower frequencies (**Table 7**). A minimum spanning tree is shown in **Figure 6**.

**Table 7:** Multilocus sequence typing (MLST) results of 41 CRPA clinical isolates.

STs	*Acsa*	*Aroe*	*Guaa*	*Mutl*	*Nuod*	*Ppsa*	*Trpe*	Frequency, % (n/N)
ST1076	5	4	57	62	1	1	26	29.3(12/41)
ST274	23	5	11	7	1	12	7	7.3(3/41)
ST1129	22	5	91	54	4	4	7	4.9(2/41)
ST1965	158	4	1	10	3	6	3	4.9(2/41)
ST4399	38	11	3	318	1	2	4	4.9(2/41)
ST4400	28	3	94	318	1	4	10	2.4(1/41)
ST1025	6	5	5	3	3	13	26	2.4(1/41)
ST532	5	4	5	5	5	20	4	2.4(1/41)
ST1706	11	6	19	3	4	4	9	2.4(1/41)
ST766	17	5	12	43	14	4	7	2.4(1/41)
ST646	11	5	6	11	2	4	19	2.4(1/41)
ST357	2	4	5	3	1	6	11	2.4(1/41)
ST938	15	20	26	13	3	64	2	2.4(1/41)
ST2069	35	5	36	72	4	42	1	2.4(1/41)
ST2424	11	5	1	3	4	6	17	2.4(1/41)
ST3630	17	182	37	261	1	7	25	2.4(1/41)
ST1417	16	10	11	85	4	4	10	2.4(1/41)
ST281	11	57	1	5	4	4	2	2.4(1/41)
ST348	22	20	11	3	3	3	7	2.4(1/41)
ST1182	5	1	109	54	1	1	47	2.4(1/41)
ST3714	11	143	5	3	1	15	112	2.4(1/41)
ST360	15	5	36	11	27	4	2	2.4(1/41)
ST2633	11	177	11	18	3	4	11	2.4(1/41)
ST463	6	5	5	3	1	6	3	2.4(1/41)
ST970	6	5	11	3	4	3	3	2.4(1/41)

#### 3.4.3 Dissemination mechanism of *bla*_NDM_

Multilocus sequence typing of the six *bla*_NDM_ -positive isolates identified six distinct sequence types (ST463, ST4400, ST646, ST357, ST532, and ST970, Refer to **Supplementary Table S5** for details.). The lack of clonal relatedness suggests that horizontal gene transfer (HGT) is the primary mechanism driving the dissemination of *bla*_NDM_ among CRPA isolates in this study.

### 3.5 Biofilm formation assay results

#### 3.5.1 Overall characteristics

**Figure 7:**
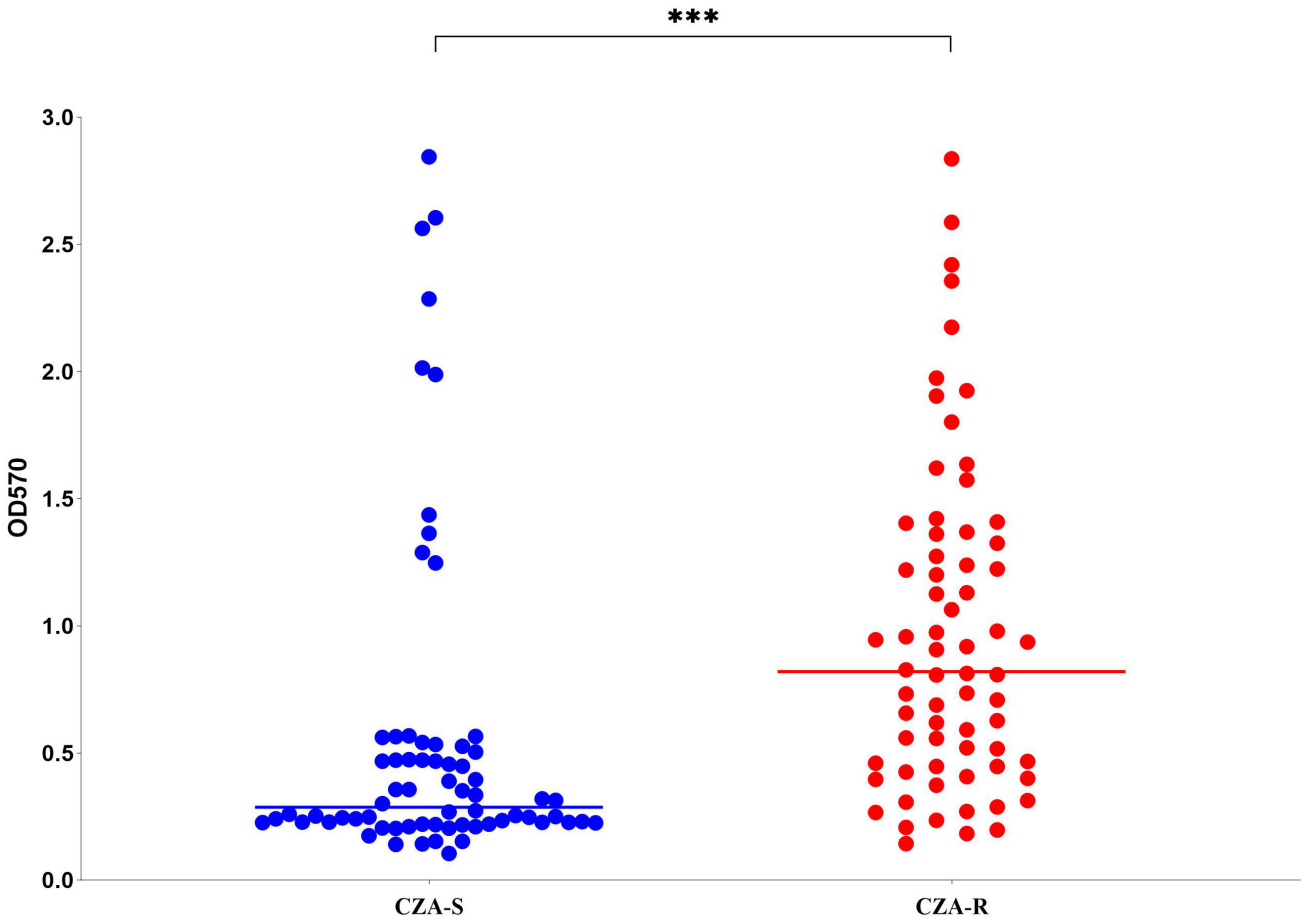
Biofilm formation (OD_570_) in 68 CZA-R and 68 CZA-S CRPA isolates. Statistical comparison was performed using the Mann-Whitney U test. SPSS 27.0. p  < <.0. (marked ***) was defined as a highly significant difference.

Biofilm formation capacity was assessed in 136 CRPA isolates (68 CZA-R and 68 CZA-S). The overall positivity rate was 97.79% (133/136). A significant difference in the distribution of biofilm formation strength was observed between the CZA-R and CZA-S groups (Z = -6.011, *p* < 0.001) (**Table 8**). Specifically, the proportion of strong positive (+++) isolates was significantly higher in the CZA-R group (66.18%, 45/68) compared to the CZA-S group (14.71%, 10/68). In contrast, the CZA-S group was predominantly composed of weak positive (+) isolates (45.59%, 31/68) and included three (4.41%) biofilm-negative isolates, which were not found in the CZA-R group. Quantitative analysis further confirmed that the median biofilm formation amount (OD_570_) was significantly higher in the CZA-R group (0.820 [IQR: 0.450–1.352]) than in the CZA-S group (0.287 [IQR: 0.226–0.521]; Z = -5.201, *p* < 0.001) (**Figure 7**).

**Table 8:** Comparison of biofilm formation capacity between CZA-resistant and CZA-susceptible CRPA isolates [n(%)].

Biofilm formation strength	CZA-R group (n=68)	CZA-S group (n=68)	Statistical value	*P* -value
Negative (–)	0 (0)	3 (4.41)	Z = -6.011	<0.001
Weak (+)	7 (10.29)	31 (45.59)		
Moderate (++)	16 (23.53)	24 (35.29)		
Strong (+++)	45 (66.18)	10 (14.71)		

The biofilm-forming capacity (categorized as: non-biofilm former (–), weak [+], moderate [++], or strong [+++]) of 68 CZA-R and 68 CZA-S CRPA was compared using the Mann-Whitney U test, a non-parametric statistical method appropriate for ordinal data.

#### 3.5.2 Characteristics of the ST1076 clone

All 12 ST1076 isolates (8 CZA-R and 4 CZA-S) were capable of forming biofilms. The biofilm formation strength was significantly higher in the CZA-R subgroup than in the CZA-S subgroup (U = 2.000, *p* = 0.012), with a higher proportion of strong positive (+++) isolates (75.0% vs. 0%).Detailed data are provided in **Supplementary Table S6**.

### 3.6 Efflux pump gene expression

**Figure 8:**
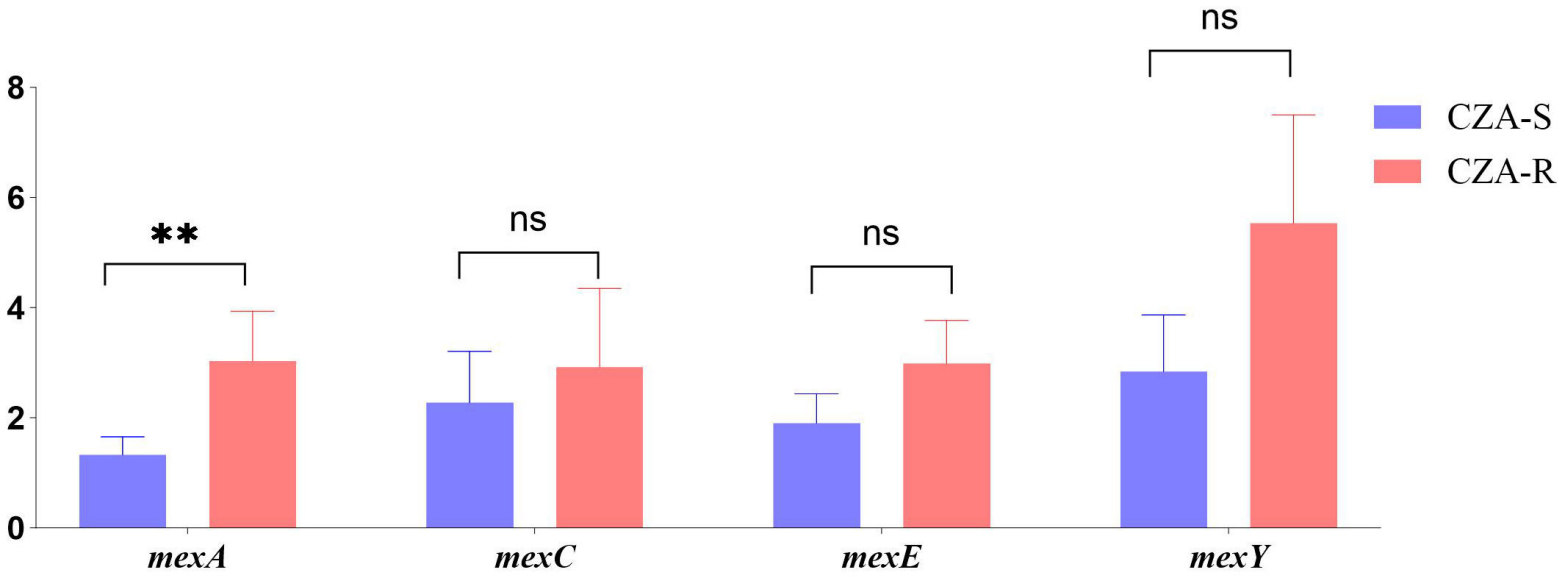
Comparative analysis of efflux pump gene expression between 12 CZA-R and 12 CZA-S CRPA isolates. Statistical comparison was performed using independent samples t-test or Mann-Whitney U test. SPSS 27.0. p < 0.05 (marked *) was defined as a significant difference.

The expression levels of four efflux pump genes (*mexA, mexC, mexE, and mexY)* were quantified using qRT-PCR in 12 CZA-R and 12 CZA-S isolates. As shown in **Figure 8**, the expression of *mexA* was significantly upregulated in the CZA-R group compared to the CZA-S group (Z = -2.656, p= 0.007). In contrast, no significant differences were observed in the expression of *mexC, mexE, or mexY* between the two groups (**Figure 8**).

### 3.7 Whole-genome sequencing analysis

**Figure 9:**
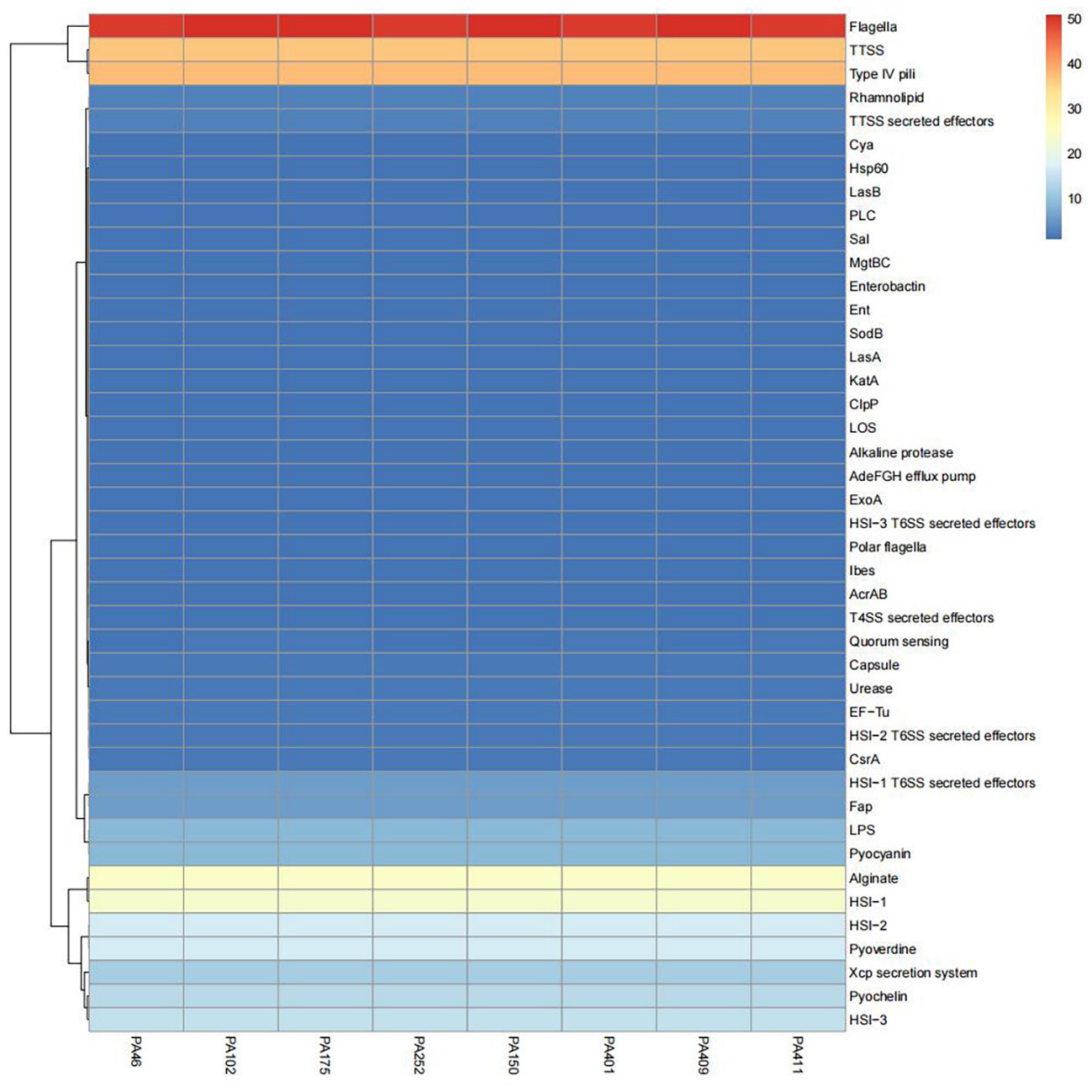
Comparative heatmap analysis of virulence-associated determinants in 8 CZA-R vs. 8 CZA-S CRPA isolates. Heatmap illustrates the presence frequency of virulence-associated factors across clinical CRPA isolates (x-axis) versus pathogenicity determinants (y-axis). Color intensity corresponds to detection frequency (blue: low, red: high).

Comparative genomic analysis was performed on eight ST1076 isolates (four CZA-R and four CZA-S). The results revealed that the profiles of 38 core virulence genes were highly conserved among the ST1076 strains, with no significant differences observed between the CZA-R and CZA-S groups (**Figure 9**).

## 4 Discussion

This study provides a systematic analysis of the prevalence, clinical risk factors, and molecular mechanisms underlying CZA resistance among carbapenem-resistant CRPA isolates in Ningbo, China. Our findings reveal a serious and multifaceted resistance problem, driven by the convergence of high-risk clones (especially ST1076), carbapenemase gene acquisition (e.g., *bla_NDM_*), enhanced biofilm formation, and efflux pump overexpression.

We report for the first time a CZA resistance rate of 24.37% among CRPA isolates in Ningbo. Although this is lower than the 28% reported by the Global *P.aeruginosa* Surveillance (GPAS) program (CM et al., 2021), it is significantly higher than the national average (18.1%) documented in the 2024 CHINET (https://www.chinets.com/Content/File/CHINET2024年全年细菌耐药监测结果.pptx) China Surveillance Report and also exceeds rates from other regions in Zhejiang Province (Yanyan et al., 2023). This suggests unique local selective pressures and/or clonal transmission. ST1076 was identified as the predominant clone, accounting for 29.3% of all isolates and 40.0% of the CZA-R group, indicating its major role in driving CZA resistance through high adaptability and nosocomial transmission. This pattern differs from clonal distributions reported in other parts of Zhejiang (Yuexing et al., 2022), highlighting the region-specific nature of resistance epidemiology.

MLST analysis confirmed the dominance of ST1076. In contrast to globally prevalent high-risk clones such as ST235 and ST111—which are often associated with virulence factors like *exoU* and high transmissibility (Felice et al., 2024)—the local ST1076 clone is characterized by enhanced biofilm formation and efflux pump overexpression, reflecting its successful regional adaptation. Whole-genome sequencing revealed that its core virulence genes were highly conserved, indicating that resistance acquisition did not compromise pathogenicity, defining it as a dangerous “resistant-adaptive” clone that demands urgent attention from clinicians and infection control teams.

CZA resistance in *P. aeruginosa* is often multifactorial (Li et al., 2024). Our results highlight the following key mechanisms:

1.Prevalence of *bla*_NDM_ and Cross-Resistance:

The *bla*_NDM_ metallo-β-lactamase gene was detected at a significantly higher rate in the CZA-R group (7.4% vs. 0.5%; *p* < 0.01). Since avibactam does not inhibit metallo-β-lactamases (such as NDM) (George et al., 2021), its presence confers intrinsic resistance to CZA. This aligns with reports that metallo-β-lactamases (including *bla*_IMP_ and *bla*_VIM_) mediate CZA resistance (Li et al., 2023; Yanyan et al., 2023), though *bla*_NDM_ remains less commonly reported in *P. aeruginosa* (J et al., 2025). Its distribution across multiple sequence types suggests horizontal gene transfer via plasmids or integrons (M et al., 2024), underscoring the need for rigorous infection control. Moreover, 50% of *bla*_NDM_ -positive isolates were resistant to aztreonam, suggesting possible co-occurrence of AmpC overexpression, efflux pump activity, or other undefined mechanisms (C et al., 2024). It should be noted that *bla*_NDM_ explains only a subset (∼7.4%) of resistant cases, indicating that other mechanisms play more dominant roles.

2.Contribution of Other Key Resistance Mechanisms:

Non-carbapenemase mechanisms also contributed significantly. Biofilm formation was significantly stronger in CZA-R isolates, acting as a physical barrier to antibiotic penetration and promoting tolerance (AJ et al., 2023; MD et al., 2024). The presence of indwelling devices (e.g., central venous catheters, drains) was an independent risk factor, corroborating the role of biofilms in treatment failure and relapse. Additionally, overexpression of *mexA* (a component of the MexAB-OprM efflux system) was observed (2.04-fold increase, *p* = 0.007). Although avibactam is not a substrate, this pump efficiently reduces intracellular concentrations of ceftazidime (Li et al., 2024), thereby diminishing CZA efficacy. Together, these mechanisms explain most non- *bla*_NDM_ -mediated resistance.

Other reported mechanisms may also be involved, such as OprD porin loss or mutation, AmpC β-lactamase hyperproduction, and activity of other efflux systems (e.g., MexXY-OprM) (Yuexing et al., 2022; B et al., 2023). These mechanisms often act synergistically—for example, efflux pump upregulation combined with porin deficiency can markedly increase resistance levels (Yuexing et al., 2022). Although not directly investigated here, their potential role warrants further study.

We observed that approximately 13% of CZA-R CRPA isolates remained susceptible to ceftazidime (CAZ) alone. This phenomenon highlights the complexity of bacterial resistance mechanisms, particularly concerning the types of enzymes inhibited by avibactam and their functional states. Multiple studies have indicated that the coexistence of CZA resistance and susceptibility to CAZ alone can often be attributed to several mechanisms: β-lactamase mutations (e.g., specific mutations in genes like *bla*_KPC-135_, which may alter the enzyme’s affinity for the inhibitor avibactam), alterations in outer membrane permeability(mutations or loss of outer membrane proteins such as LamB may reduce the efficiency of avibactam entry into the bacterial cell, while having a lesser impact on the penetration of ceftazidime alone), and changes in enzyme expression levels (e.g., upregulation of genes like *bla*_KPC_ in some strains, which can mediate resistance to CZA).

### 4.1 Clinical implications of cross-resistance for empirical therapy in critically ill patients

Given that 40.50% of isolates were from the ICU—where CRPA commonly causes life-threatening infections such as ventilator-associated pneumonia and bloodstream infections (C et al., 2025)—empirical treatment strategies are of utmost importance. Our findings reveal a troubling cross-resistance profile: CZA-R isolates exhibited co-resistance to meropenem (94.12%), ceftazidime (86.76%), piperacillin/tazobactam (88.24%), cefoperazone/sulbactam (85.29%), and fluoroquinolones (e.g., 50.00% to ciprofloxacin). The additional resistance to aztreonam (50%) among *bla*_NDM_ -positive isolates further limits therapeutic options.

In this high-resistance setting, empirical use of CZA for suspected CRPA infections—especially in units with known ST1076 transmission or high *bla*_NDM_ rates—carries a high risk of failure. For critically ill patients with risk factors (e.g., recent antibiotic exposure, invasive procedures), alternative agents with activity against metallo-β-lactamase-producing strains should be considered, such as cefiderocol, imipenem-cilastatin-relebactam, or innovative combination regimens (e.g., high-dose aztreonam with avibactam) (R et al., 2022). Rapid molecular diagnostics are essential for de-escalation and targeted therapy. The strong biofilm and efflux pump phenotypes also support the use of non-systemic strategies (e.g., antimicrobial-coated devices, local irrigation) and prompt removal of infected hardware.

### 4.2 Limitations

This study has several limitations. Its single-center design may affect generalizability; multi-center validation is needed. The WGS cohort was relatively small—expanding sample size in future studies would improve the resolution of genetic determinants of resistance and virulence.

## 5 Conclusion

This study illuminates the high prevalence and complex mechanistic landscape of CZA resistance among CRPA isolates in Ningbo. Key findings include: 1) a resistance rate significantly above the national average, linked to clonal expansion of ST1076; 2) multifactorial resistance mechanisms involving *bla*_NDM_, enhanced biofilm formation, and MexAB-OprM overexpression; and 3) extensive cross-resistance narrowing therapeutic options.

These insights have immediate clinical implications: CZA should not be used empirically in high-risk settings without susceptibility confirmation; novel anti-pseudomonal agents and rapid diagnostics should be prioritized; and enhanced infection control measures are urgently needed to contain the spread of resistant clones and mobile genetic elements. Future studies should focus on larger-scale surveillance, mechanistic dissection of resistance synergy, and clinical evaluation of newer therapeutic regimens in this population.

## Data Availability

The raw data supporting the conclusions of this article will be made available by the authors, without undue reservation.
