# Supplementary material for: Study on resistance mechanisms and molecular epidemiology of carbapenem-resistant Pseudomonas aeruginosa to ceftazidime/avibactam in a certain region of China
Source: Front Cell Infect Microbiol. 2025 Oct 21;15:1643755. doi: 10.3389/fcimb.2025.1643755 (PMC12583027; doi:10.3389/fcimb.2025.1643755)
Supplement: Supplementary file 1 [file Table1.docx]

**Table S1. Reference ranges for antimicrobial susceptibility testing results by VITEK 2​**​

| **Antimicrobial Agent** | **Testing Method** | **Breakpoints** † |
| --- | --- | --- |
| Aztreonam | Disk Diffusion | R ≤ 15, S ≥ 22 |
| Cefoperazone/Sulbactam | MIC | S ≤ 16, R ≥ 64 |
| Cefepime | MIC | S ≤ 8, R ≥ 32 |
| Tobramycin | MIC | S ≤ 1, R ≥ 4 |
| Amikacin | MIC | S ≤ 16, R ≥ 64 |
| Ciprofloxacin | MIC | S ≤ 0.5, R ≥ 2 |
| Piperacillin/Tazobactam | MIC | S ≤ 16/4, R ≥ 64/4 |
| Imipenem | MIC | S ≤ 2, R ≥ 8 |
| Levofloxacin | MIC | S ≤ 1, R ≥ 4 |
| Meropenem | MIC | S ≤ 2, R ≥ 8 |
| Ceftazidime | MIC | S ≤ 8, R ≥ 32 |
| Ticarcillin/Clavulanate | MIC | S ≤ 16, R ≥ 128 |
| Colistin‡ | MIC | I ≤ 2, R ≥ 4 |

† ​​Breakpoint units:​​ Disk diffusion (K-B method) results are interpreted based on zone diameter in ​​millimeters (mm)​​; broth microdilution (MIC method) results are interpreted based on ​​minimum inhibitory concentration in μg/mL​​.

‡For colistin, a loading dose should be administered followed by a maximum dose adjusted for renal function (refer to international consensus guidelines). MIC: Minimum Inhibitory Concentration; R: Resistant; S: Susceptible; I: Intermediate.

**Table S2. Primer sequences for efflux pump genes and the reference gene.​**

| **Gene** | **Direction** | **Sequence (5’–3’)** |
| --- | --- | --- |
| *mexA* | Forward | AACCCGAACAACGAGCTG |
|  | Reverse | ATGGCCTTCTGCTTGACG |
| *mexC* | Forward | GTACCGGCGTCATGCAGGGTTC |
|  | Reverse | TTACTGTTGCGGCGCAGGTGACT |
| *mexE* | Forward | CCAGGACCAGCACGAACTTCTTGC |
|  | Reverse | CGACAACGCCAAGGGCGAGTTCACC |
| *mexY* | Forward | TCGCCCTATTCCTGCTG |
|  | Reverse | AGTTCGCTGGTGATGCC |
| *rpoD* | Forward | GGGCTGTCTCGAATACGTTGA |
|  | Reverse | ACCTGCCGGAGGATATTTCC |

​**​Table S3. Comparison of Antibiotic Resistance Profiles between CZA-Resistant and CZA-Susceptible CRPA Isolates [n(%)]​**

| **Antibiotic** | **CZA-R Group (n=68)** | **CZA-S Group (n=211)** | **χ² Value** | ***p*-Value** |
| --- | --- | --- | --- | --- |
| Ceftazidime | 59 (86.76) | 149 (70.62) | 7.069 | 0.008 |
| Cefepime | 44 (64.71) | 120 (56.87) | 1.303 | 0.262 |
| Aztreonam | 62 (91.18) | 164 (77.73) | 6.047 | 0.014 |
| Meropenem | 64 (94.12) | 175 (82.94) | 5.233 | 0.022 |
| Imipenem | 65 (95.59) | 202 (95.73) | <0.001 | >0.999 |
| Tobramycin | 11 (16.18) | 10 (4.74) | 9.665 | 0.002 |
| Amikacin | 6 (8.82) | 3 (1.42) | 6.810 | 0.009 |
| Ciprofloxacin | 34 (50.00) | 66 (31.28) | 7.837 | 0.005 |
| Levofloxacin | 43 (63.24) | 109 (51.66) | 2.779 | 0.096 |
| Colistin | 3 (4.41) | 6 (2.84) | 0.058 | 0.809 |
| Ticarcillin/Clavulanate | 60 (88.24) | 168 (79.62) | 2.555 | 0.110 |
| Piperacillin/Tazobactam | 60 (88.24) | 157 (74.41) | 5.689 | 0.017 |
| Cefoperazone/Sulbactam | 58 (85.29) | 141 (66.82) | 8.578 | 0.003 |

Note:​​ Data are presented as number of resistant isolates with percentage in parentheses [n(%)]. χ² test was used for comparisons between groups. p-values < 0.05 were considered statistically significant.

**Table S4. Prevalence of MDR-CRPA among CRPA isolates​​[n(%)]​**

| **Group** | **CZA-R Group (n=68)** | **CZA-S Group (n=211)** | **χ²** | ***p*-Value** |
| --- | --- | --- | --- | --- |
| MDR-PA | 64 (94.12) | 176 (83.41) | 4.901 | 0.027 |
| No-MDR-PA | 4 (5.88) | 35(16.59) |  |  |

Note:​​ The χ² test was used for comparison between groups. p-value < 0.05 was considered statistically significant.

**Table S5. Association between sequence types and CZA susceptibility profiles of blaNDM -positive isolates​**

| **Sequence Type (ST)** | **Resistance Phenotype** | **Frequency, % (n/N)** |
| --- | --- | --- |
| ST463 | CZA-R | 16.7 (1/6) |
| ST4400 | CZA-R | 16.7 (1/6) |
| ST646 | CZA-R | 16.7 (1/6) |
| ST357 | CZA-R | 16.7 (1/6) |
| ST532 | CZA-R | 16.7 (1/6) |
| ST970 | CZA-S | 16.7 (1/6) |

​**​****Table S6. Biofilm formation capacity of 12 ST1076 isolates​​[n(%)]​**

| **Biofilm Intensity** | **CZA-R Group (n=8)** | **CZA-S Group (n=4)** | **Statistical Value** | ***p*-value** |
| --- | --- | --- | --- | --- |
| Negative (-) | 0(0%) | 0(0%) | -2.596 | 0.012 |
| Weak (+) | 0(0%) | 2(50%) |  |  |
| Moderate (++) | 2(25%) | 2(50%) |  |  |
| Strong (+++) | 6(75%) | 0(0%) |  |  |
